# Hyperspectral Data and Machine Learning for Estimating CDOM, Chlorophyll *a*, Diatoms, Green Algae and Turbidity

**DOI:** 10.3390/ijerph15091881

**Published:** 2018-08-30

**Authors:** Sina Keller, Philipp M. Maier, Felix M. Riese, Stefan Norra, Andreas Holbach, Nicolas Börsig, Andre Wilhelms, Christian Moldaenke, André Zaake, Stefan Hinz

**Affiliations:** 1 Institute of Photogrammetry and Remote Sensing, Karlsruhe Institute of Technology, Kaiserstr. 12, 76131 Karlsruhe, Germany; philipp.maier@kit.edu (P.M.M.); felix.riese@kit.edu (F.M.R.); stefan.hinz@kit.edu (S.H.); 2 Institute of Applied Geoscience, Karlsruhe Institute of Technology, Kaiserstr. 12, 76131 Karlsruhe, Germany; stefan.norra@kit.edu (S.N.); nicolas.boersig@kit.edu (N.B.); andre.wilhelms@kit.edu (A.W.); 3 Department of Bioscience, Aarhus University, Frederiksborgvej 399, 4000 Roskilde, Denmark; anho@bios.au.dk; 4 bbe Moldaenke GmbH, Preetzer Chaussee 177, 24222 Schwentinental, Germany; CMoldaenke@bbe-moldaenke.de (C.M.); AZaake@bbe-moldaenke.de (A.Z.)

**Keywords:** machine learning, regression, water quality parameters, hyperspectral data, spectral features, algae, chlorophyll *a*, multi-sensor system, fluorometer, field campaign

## Abstract

Inland waters are of great importance for scientists as well as authorities since they are essential ecosystems and well known for their biodiversity. When monitoring their respective water quality, in situ measurements of water quality parameters are spatially limited, costly and time-consuming. In this paper, we propose a combination of hyperspectral data and machine learning methods to estimate and therefore to monitor different parameters for water quality. In contrast to commonly-applied techniques such as band ratios, this approach is data-driven and does not rely on any domain knowledge. We focus on CDOM, chlorophyll *a* and turbidity as well as the concentrations of the two algae types, diatoms and green algae. In order to investigate the potential of our proposal, we rely on measured data, which we sampled with three different sensors on the river Elbe in Germany from 24 June–12 July 2017. The measurement setup with two probe sensors and a hyperspectral sensor is described in detail. To estimate the five mentioned variables, we present an appropriate regression framework involving ten machine learning models and two preprocessing methods. This allows the regression performance of each model and variable to be evaluated. The best performing model for each variable results in a coefficient of determination $R^{2}$ in the range of 89.9% to 94.6%. That clearly reveals the potential of the machine learning approaches with hyperspectral data. In further investigations, we focus on the generalization of the regression framework to prepare its application to different types of inland waters.

## 1. Introduction

Inland waters are important ecosystems and biodiversity hotspots as well as prominent water resources for diverse human needs [1,2]. When monitoring the water quality of inland waters, precise data about the area-wide distribution of water quality parameters is crucial. By monitoring several water quality parameters such as colored dissolved organic matter (CDOM), chlorophyll *a*, green algae, diatoms and turbidity, conclusions about water quality can be drawn. In this contribution, we refer to the three water parameters CDOM, chlorophyll *a* and turbidity, as well as the quantity of the two algae types, diatoms and green algae, as water quality parameters for reasons of simplicity.

CDOM mainly consists of fulvic and humic substances, which strongly absorb radiation in the ultraviolet spectrum. This absorption results in water colorings from yellow to dark brown. In high concentrations, CDOM can influence bio-optical properties of surface waters and can cause large variations in the penetration of radiation [3]. Chlorophyll as a group of pigments is involved in all phototrophic organisms including algae and some species of bacteria. Chlorophyll *a* is a prominent pigment, since almost all phototrophic organism contain it [4]. Measuring and monitoring the chlorophyll *a* concentration provides insight into the phytoplankton biomass, as well as their trophic state [5]. For example, surface waters that show high chlorophyll *a* concentrations simultaneously contain high levels of nutrients, which in general, are phosphorus and nitrogen. Therefore, chlorophyll *a* can be utilized as an indirect indicator of nutrient levels. Additional to chlorophyll *a*, green algae contains beta-carotene as pigments. This algae species is characterized by a variety of unicellular species. Diatoms are unicellular micro-algae and are a main component of phytoplankton. Diatoms represent a main primary producer of organic substances, and thus, they function as an essential part of the food web of aquatic systems [6]. The relative composition of diatoms serves as an indicator for the degree of eutrophication, the diatom index and other water parameters such as pH value or salinity [7]. Turbidity measures to which extent light is scattered and absorbed. A large amount of suspended solids leads to high turbidity. High turbidity affects water quality and aquatic life due to the blocking of sunlight. This decreases the primary production (of phytoplankton), as well as the transport and possible release of pollutants [8].

Conventional monitoring techniques measure water quality parameters point-based either in situ or later in a laboratory. These measurements are precise at a specific location and provide a detailed depth-profile. When covering large water areas, they are spatially limited, costly and time-consuming [9].

A complementary solution to expensive in situ measurements arises in the field of remote sensing. For example, satellite-based measurements are quite common in monitoring oceans, respectively ocean color [10]. At the same time, however, remote sensing techniques and methods for monitoring inland waters have made slow progress since the installed satellite missions are predestined to measure ocean colors. The inhomogeneity of inland waters impedes the retrieval of physical, biological and chemical water properties [9]. In the last decade, the advanced developments in the field of hyperspectral remote sensing have opened up innovative data acquisition. Hyperspectral cameras are characterized by a high spectral resolution, which is predestined to evaluate water quality parameters when covering the respective wavelength range from 450 nm to 950 nm. In general, a hyperspectral camera records the surface reflectance of the water components. In the following, we refer to the reflectances of different water quality parameters as their respective spectral signatures.

The spectral signature of chlorophyll *a* in water contains a reflectance minimum at about 670 nm and reflectance maxima in the green and red spectral range [11]. To determine CDOM content, chlorophyll *a* concentrations and turbidity in the field of multispectral remote sensing, recent empirical methodologies mainly have relied on the engineering features by selecting specific spectral bands [12,13,14,15,16,17]. With respect to the examination of different algae, brown, green, blue-green and red algae species based on hyperspectral spectra, an experimental study has been published as a methodological basis to monitor changes of phytoplankton via remote sensing [18]. In addition to the feature engineering approaches of the near-infrared (NIR) and red spectral range, approaches calculating the derivatives near 690 nm are suitable for identifying high chlorophyll *a* concentrations from spectral data [13].

The estimation of water quality parameters with high-dimensional spectral data is a non-linear regression problem. Data-driven machine learning approaches, in general, should be able to handle complex problems without any domain knowledge (no need for a physical model) when accessing sufficient input data [19]. Only a few studies have focused on applying machine learning to estimate CDOM and chlorophyll *a* concentrations. CDOM can be estimated for example based on either functional linear models [20] or linear stepwise regression [21]. In our previous study, we introduced a regression framework involving five machine learning models to estimate water quality parameters with hyperspectral data [22]. The suitability of machine learning models in this context has been shown clearly.

In this paper, we rely on a multi-sensor dataset, which has been measured under real-world conditions on the river Elbe, Germany. This way, we ensure precise monitoring and the ability to transfer our applied methodology. CDOM, chlorophyll *a*, green algae, diatoms and turbidity have been monitored precisely with two different sensors. These measurements function as reference data. Hyperspectral snapshot data has been recorded and function as input data for the machine learning models. In contrast to Maier and Keller [22], we evaluate a more sophisticated framework containing ten distinct machine learning models and two preprocessing methods. This framework has been applied and evaluated in the context of non-linear regression problems with hyperspectral input data and environmental target variables [23]. The main objectives of this paper are:

- a detailed description of our measurements of water quality parameters with the Biofish multi-sensor system and the PhycoSens fluorometer, which is yet non-existent;
- a comprehensive analysis of the potential of an appropriate regression framework based on different regression models, e.g., linear models, tree ensemble methods and artificial neural networks;
- an underlying analysis of two distinct preprocessing methods combined with a detailed evaluation of the regression performance;
- a detailed visualization of the regression results compared to the real probe measurements based on recorded GPS tracks along the river Elbe.

In Section 2, the sensor systems and the measurement setup during the Elbe field campaign are introduced. We also describe the dataset itself, which is used for the estimation of the five water quality parameters based on a regression framework. The workflow of the framework is illustrated in Section 3. The regression results are presented in Section 4. Afterwards, we evaluate and assess the regression performance of the ten machine learning models combined with the preprocessing steps in Section 5. In Section 6, the underlying study is concluded, combined with an outlook of future studies.

## 2. Sensors and Datasets

**Figure 1:**
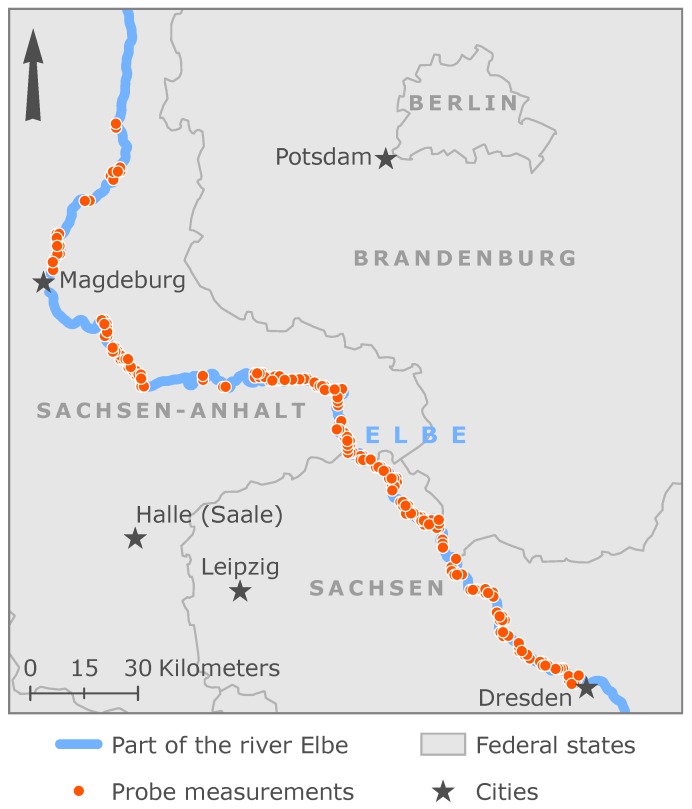
Map of the study area with the probe measurements.

We rely on a multi-sensor dataset to analyze the potential of the regression framework to estimate the concentrations of CDOM, chlorophyll *a*, green algae, diatoms and turbidity. The dataset was measured in a field campaign during the Elbe field campaign. It was carried out on the research vessel Elbegrund of the German Federal Waterways and Shipping Administration of Germany. We monitored the river Elbe in Germany along a 575 km stretch from Bad Schandau downstream to Geesthacht from 24 June–12 July 2017. The fluorometer PhycoSens, the Biofish multi-sensor system and a visible and near-infrared (VNIR) Cubert hyperspectral sensor were the applied sensor systems. Figure 1 shows the map of the study area and the area of the probe measurements. In the following subsections, we describe the measurement system and the respective data acquisition of each sensor.

### 2.1. Sampling Chlorophyll a, Green Algae and Diatoms

The PhycoSens fluorometer is mounted in front of the research vessel. This instrument enables in situ measurements of water quality parameters without additional sample preparation by filtration or with solvent. It simultaneously determines chlorophyll *a* concentrations, transmission of light and optional photosynthetic activity. This sensor also measures the amount of unbound phycocyanin, which mirrors the release of blue-green algae contents. Chlorophyll *a* and phycocyanin content is excited by seven LEDs at frequencies of 370 nm, 430 nm, 470 nm, 525 nm, 590 nm and 610 nm to obtain a meaningful fluorescence excitation spectrum. The fluorescence emission is measured as an answer to the excitation and allocated to the different algae classes such as green algae, cyanobacteria, cryptomonads or the class of diatoms. In this paper, we rely on the PhycoSens sensor to measure chlorophyll *a*, green algae and diatom concentrations every five minutesin µg/L. In this context, the green algae and diatom concentrations are expressed as the chlorophyll *a* equivalent concentrations derived from specific fluorescence signatures of green algae.

### 2.2. Sampling CDOM and Turbidity

The multi-sensor system Biofish monitors eight relevant water quality parameters: temperature, electrical conductivity, oxygen concentration and saturation, pH, CDOM, chlorophyll *a*, turbidity and photosynthetically active radiation. Although the Biofish sensor system also samples the chlorophyll *a* concentration, we rely on the PhycoSens chlorophyll *a* measurements in this paper. Comparisons between the two sensors will be addressed in further studies. All data was sampled online at a 4 Hz frequency and were tagged immediately with GPS measurements. Detailed sensor specifications are presented in the Supporting Information report [24]. The Biofish sensor system is deployed from a boat and can be operated in different modifications, which range from 2D–3D spatial measurements.

**Figure 2:**
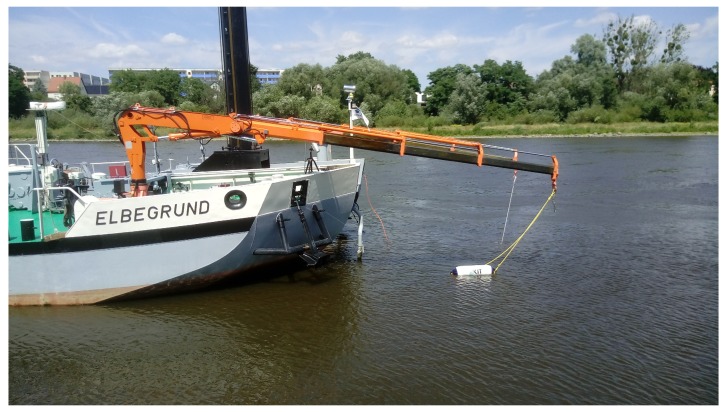
Application of the Biofish sensor system (white floating cylinder) from the research vessel Elbegrund in the river Elbe.

During the Elbe field campaign, the Biofish sensor system was installed at a fixed depth of around 0.5 m underneath a floating cylinder and was mounted on a crane in front of the research vessel (cf. Figure 2). The monitoring is configured to run in the 2D spatial resolution mode. To evaluate the data, we calculate median values of each parameter for every recorded minute. In this study, we rely on the two water quality parameters CDOM in ${\mathrm{ppb}}_{\mathrm{QS}}=10^{-9}$ and turbidity in Formazin Turbidity Unit (FTU) measured by the Biofish sensor system.

### 2.3. Recording Hyperspectral Images

The hyperspectral snapshot sensor Cubert UHD 285 records high-dimensional images non-invasively every 0.5 min to 1 min. It was mounted on a tripod next to the Biofish sensor system at the front of the research vessel. The calibration and measurement setup of the hyperspectral sensor was described in our previous studies [22,25]. Each hyperspectral image was characterized by 50×50 pixels and 125 spectral channels, each with a spectral resolution of 4 nm. The spectrum ranged from 450 nm to 950 nm. We select an area in each image which was free of bubble formations, shadows or waves to calculate a mean spectra per image manually. To exclude sensor errors, we applied a feature band selection resulting in a range of wavelengths between 470 and 910 nm.

### 2.4. Elbe Field Campaign Datasets

For a temporal matching of the sampled PhycoSens data to the hyperspectral data, we need to extend the former by a linear interpolation. This was possible due to the continuous change of the sampled chlorophyll *a*, green algae and diatoms concentrations. The data sampled by the Biofish sensor system can be matched directly to the hyperspectral data due to its continuously high temporal resolution.

**Figure 3:**
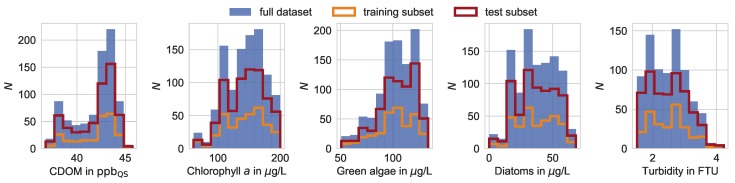
Distributions of the water quality parameter values. Each full dataset (blue bars) is split randomly into a training (orange) and a test (red) subset. The number of datapoints is symbolized as *N*.

The Elbe field campaign resulted in five datasets, one dataset for each of the five water quality parameters. A dataset contains datapoints, whereby one datapoint is defined by 111 selected hyperspectral bands and one value of a respective water quality parameter. We performed all regressions with the hyperspectral data as input data and the respective water quality parameter as the target variable. Outliers of the target variable (histogram bin content >3) were excluded, as described in the following, to ensure an appropriate regression performance of the framework. In detail, we dismissed chlorophyll *a* concentrations above 200 µg/L and diatom concentrations above 70 µg/L, and we included green algae concentrations exclusively in the range of 40 µg/L to 140 µg/L. Each full dataset was randomly divided into a training and test subset to prevent overfitting of the dataset and to meet adequate conventions in the context of machine learning. Table 1 shows the distribution of counts between the five datasets of each water quality parameter. Furthermore, the distributions of the water quality parameter as the target variable of the regression are illustrated in Figure 3.

## 3. Methodology

**Figure 4:**
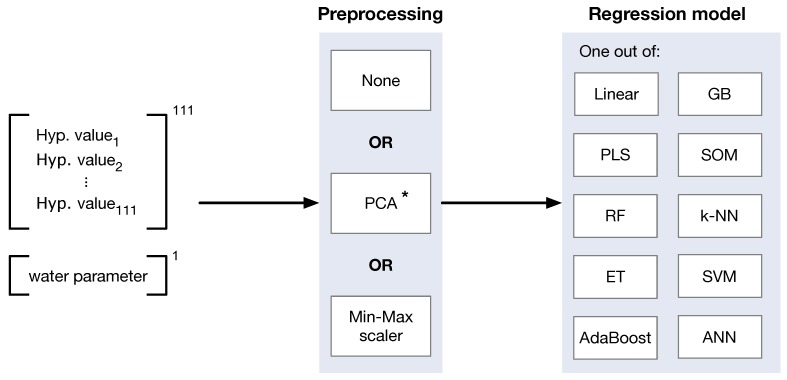
Schematic representation of the regression framework (adapted from [23]). The water quality parameter is either CDOM, chlorophyll *a*, green algae, diatoms or turbidity. * PCA is applied solely on the hyperspectral vector data.

The applied regression framework consists of two distinct steps: the preprocessing and the regression model to estimate the five water quality parameters. Figure 4 illustrates the schema of the regression framework.

### 3.1. Preprocessing

We applied two different preprocessing methods to enhance the regression process with high-dimensional data (cf. Figure 4, box in the middle). Principal Component Analysis (PCA) represents the first method. It decreases the dimensionality of the hyperspectral data. In this case, the first eight principal components were used instead of the full 111 features of the hyperspectral input data for the regression. These eight components were chosen, since they covered 99.9% of the variances. As a second preprocessing method, we relied on a min-max scaling. This scaling was performed for each hyperspectral band *i* individually according to the following equation:

(1) $$\begin{array}{c} \Delta_{\mathrm{scaled}}\left(x\right)=\frac{x-\min(X_{\mathrm{train},i})}{\max\left(X_{\mathrm{train},i}\right)-\min\left(X_{\mathrm{train},i}\right)}\;, \end{array}$$

with a training dataset $X_{\mathrm{train},i}$ of hyperspectral band *i*. This transformation is applied to all hyperspectral values *x* of the training and the test datasets, resulting in normalized values in the range of 0–1. The scaled test dataset might contain values <0 and >1, since it is scaled based on the training dataset. This practice is necessary to ensure that the test dataset is only used for the evaluation of the framework. We refer to the regression without any preprocessing as the baseline.

### 3.2. Regression Models

In order to estimate the five selected water quality parameters, we relied on a framework containing appropriate machine learning models (c.f. [23]). Figure 4 (right box) lists the included machine learning models: linear regression (least squares), partial least squares (PLS), random forest (RF [26]), extremely randomized trees (ET [27]), adaptive boosting (AdaBoost [28]), gradient boosting (GB [29]), k-nearest-neighbors (k-NN [30]), support vector machines (SVM [31]), artificial neural networks (ANN [32]) and a framework of self-organizing maps (SOM [33,34]). The majority of the machine learning models are implemented in the well-known Python package scikit-learn [35]. Exceptions are the ANN implemented in Tensorflow [36] and our own SOM implementation, which was explained comprehensively in [33], including the training and evaluation of the SOM. The models were selected based on the relevance in hyperspectral remote sensing. Deep neural networks [37] were not considered within the scope of this publication.

All regression models were trained in a training phase on the training subsets. During this phase, the hyperspectral data was linked to the respective water quality parameter. Therefore, the model parameters of each model were adapted to the data of the training subsets. Most regressors are supervised learners. Exclusively, the SOM framework consists of an unsupervised SOM and a supervised SOM (cf. [33]).

In contrast to model parameters, hyperparameters of the regressors need to be tuned before the training phase to improve the regression performance. A complete summary of the regression framework’s hyperparameters was presented in [23]. Except for the linear regression model, the RF and the ET, the hyperparameters of the other regression models were tuned by a basic grid search approach. Since the RF and the ET models have performed well without tuning in [38], we also relied on that setup in this contribution.

The mentioned grid search was performed independently for each model, for each water quality parameter and for each of the three preprocessing steps with 5-fold cross-validation on the training subset. The grid search optimizes the respective regressions with respect to the coefficient of determination $R^{2}$. All ANNs contain several hidden layers with 32–256 nodes per layer optimized with the Adam optimizer [39]. The best-performing topology of each ANN model was obtained by a grid search. As a result of this optimization, all ANN models trained with the PCA-preprocessed data contained one hidden layer with 64 nodes. Beyond that, we noticed no further correlation between the ANN topology and the input setup as a combination of the water quality parameter and the preprocessing method.

After successfully completing the tuning process and the training phase, the test phase started. During this, the trained regression framework estimated the selected water quality parameter with hyperspectral data. The estimated water quality parameter values were then compared to the respective measured values of the water quality parameters. The regression performance is expressed as the root mean squared error (RMSE) and the coefficient of determination $R^{2}$. Since machine learning models depend on randomization (e.g., ET), we calculated both performance measures as arithmetic means of seven independent regression runs initialized by different random seeds. Thus, we achieved reliable and robust regression results.

## 4. Results

In this section, we focus on the performance of the regression framework to estimate the five water quality parameters, the impacts of the two preprocessing methods, and the comparison between the estimated water quality parameter values and the measured ones. The regressions results for the estimation of CDOM, chlorophyll *a*, green algae, diatoms and turbidity in combination with preprocessing methods are summarized in Table 2. Among all models, ET, SVM and ANN achieved the best regression result for the five water quality parameters. The two boosting models AdaBoost and GB resulted in moderate regressions. The regression framework estimates CDOM and chlorophyll *a* with an $R^{2}$ larger than 90%. The $R^{2}$ score of green algae, diatoms and turbidity was in the range of 80–90%.

With respect to the estimation of CDOM, nearly every model delivered very good results. The best performance of $R^{2}=94.6$% was achieved by ET with a PCA preprocessing. With $R^{2}=93.7$%, the ANN with min-max scaling as the preprocessing method showed the best results among the models with this preprocessing method. In contrast, the ANN model estimated CDOM insufficiently ($R^{2}=50.8$%) with PCA-preprocessed data despite tuning efforts. Effects which seemed to emerge in this context need to be further investigated. Considering only the estimations with the baseline data, SVM performed best with $R^{2}=91.5$%.

Without any preprocessing, SVM represented the best regressor in estimating chlorophyll *a* with $R^{2}=88.0$%. The overall best results of $R^{2}=91.4$% were achieved by ET in combination with PCA. Analogous to the estimation of CDOM, the ANN model handled min-max scaled input data the best and reached $R^{2}=89.3$% for chlorophyll *a*.

On average, the regression performance of estimating green algae and diatoms was worse compared to the estimation of chlorophyll *a*. For the estimation of diatoms, SVM was again the best regressor without any preprocessing. Relying on PCA preprocessing, ANN and ET outperformed the other models. Performing the regression with min-max scaled input data, ANN once again stood out.

As for the estimation of turbidity, SVM represented the best regressor without preprocessing. ET and ANN achieved nearly an $R^{2}$ of 90% with PCA, which was the overall best performance for turbidity estimation. Furthermore, the ANN model scored $R^{2}=80.8$% with min-max scaling.

Overall, SVM represented the best regressor for baseline input data and linear regression. PLS, RF, ET, AdaBoost and GB performed better with PCA as preprocessing. In general, the performance of the SOM framework was independent of any preprocessing.

**Figure 5:**
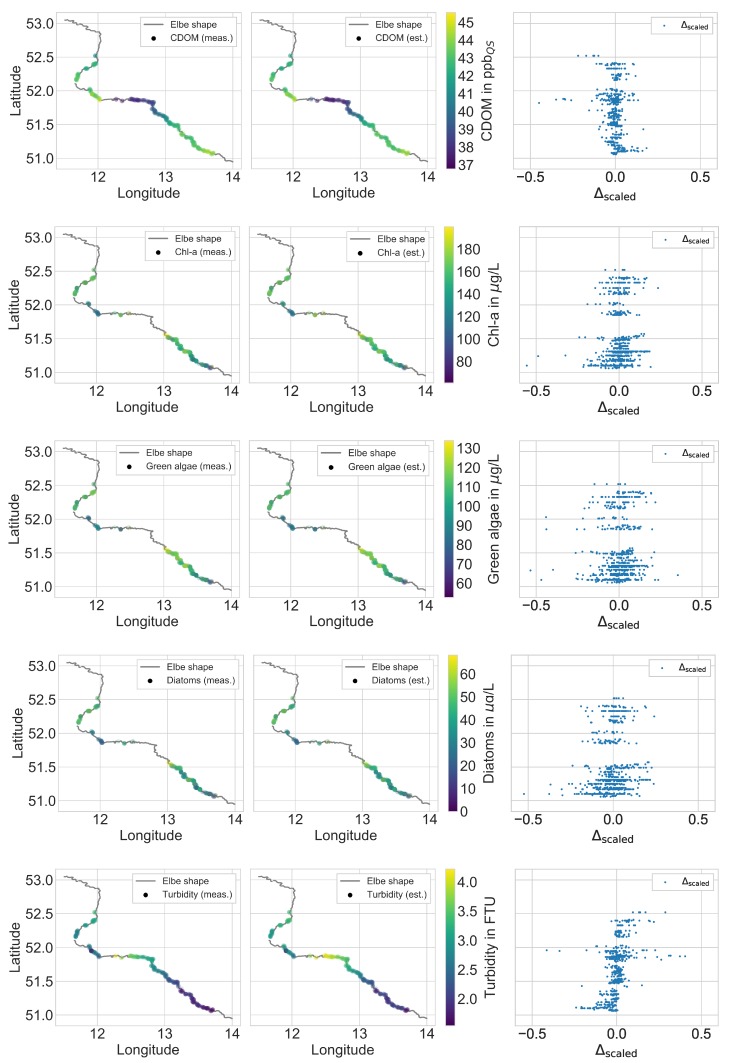
Visualization of the regression results generated by the ET model (central columns) compared to the real probe measurements (left columns) matched with their respective recorded GPS data along the river Elbe. The min-max scaled deviations $\Delta_{\mathrm{scaled}}$ between the measured (meas.) and the estimated (est.) values of the water quality parameters are illustrated in the right columns. We refer to the chlorophyll *a* concentration in this plot as Chl-a.

Figure 5 exemplifies the regression results of the ET model compared to the real probe measurements matched with their respective recorded GPS data along the Elbe. In addition, plots in the right columns represent the min-max scaled deviation $\Delta_{\mathrm{scaled}}$ between the measured and the estimated values. The scaled deviation $\Delta_{\mathrm{scaled}}$ allowed the comparison of the estimation performance of all water quality parameters. We define $\Delta_{\mathrm{scaled}}$ according to Equation (1) with a modified basic set of all squared estimation errors. Eventually, this results in a measure that is independent of the unit and the range of the target variable.

Regarding the estimation of chlorophyll *a*, the ET model underestimated the chlorophyll *a* concentration at the begin of the Elbe field campaign (cf. Figure 5, second row). Over the central profile and the end of the field campaign, an overestimation occurred. With respect to the green algae and diatom concentrations (cf. Figure 5, third and fourth row), similar conditions in the course of the Elbe field campaign can be deduced regarding the over- and under-estimation of the regressor. As for the turbidity (cf. Figure 5, last row), the models underestimated this parameter at the beginning of the field campaign. Later along the track of the Elbe, it changed to an overestimation.

**Figure 6:**
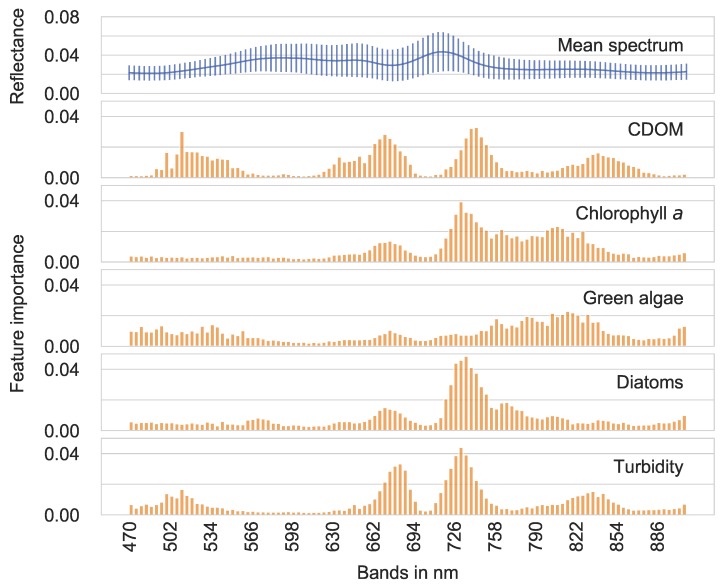
Feature importance of the ET regressor without preprocessing (baseline). The upper plot represents the mean spectrum of the hyperspectral data.

In Figure 6, we show the feature importance distributions for the hyperspectral input data of all water quality parameters generated by ET without preprocessing. Additionally, the hyperspectral mean spectrum with the standard deviation is included. To derive statements of the hyperspectral input data, we needed a feature importance distribution of the raw bands without preprocessing. Furthermore, we chose the ET regressor due to its good performance in the baseline mode. Except for the green algae distribution, the four other distributions were characterized by similar peaks: one larger peak at around 735 nm and one smaller peak at around 680 nm. The feature importance of lower wavelength ranges (less than 630 nm) and longer wavelength ranges (greater than 790 nm) was distributed randomly.

## 5. Discussion

The main methodological objective of this paper is to investigate the potential of estimating five different water quality parameters given only measured, and therefore sparse, input data. Little attention has been paid to the application of machine learning in the context of estimating water quality parameters with hyperspectral data in previous studies so far. Machine learning offers the benefit to perform regressions without possessing further knowledge of the water body or water quality parameters that are investigated. Furthermore, this approach is purely data-driven without the need to engineer new features based on domain knowledge, such as band-ratio approaches.

In Section 5.1, we first discuss the performance and applicability of the regression framework in general. We provide a summary of the essential findings regrading the different framework’s configurations. Subsequently, the feature importance of the ET regressor example without preprocessing is considered in detail in Section 5.2.

### 5.1. Performance and Applicability of the Regression Framework

In general, the regression results indicate the adequate applicability of the framework when estimating the five water quality parameters based on hyperspectral data. The framework performs without the occurrence of major systematic errors. This finding is emphasized by the random distribution of the deviations for all water quality parameters in Figure 5 (right column).

With respect to the preprocessing methods, ANN and SOM models improve their performance with min-max scaled data due to the linkage between the integrated distance measure and the normalized data. The best ANN regression results are generated when using the min-max scaled, normalized input data. This effect is plausible, since the ANN model usually weights the input data according to their values.

To increase the focus on the investigation of preprocessing methods with hyperspectral data, we might consider a combination of min-max scaling and PCA. Furthermore, a more sophisticated tuning of hyperparameters with a combined preprocessing could slightly improve the already good regression results.

Summarizing, all five water quality parameters are estimated well by different regression models. This is indicated by the best results of $R^{2}$ in the range of 89.9% to 94.6%, The good performance is a consequence of, among others, the random split between training and test subsets. Since the water quality parameters are measured as time series, each subset might contain values in close time range to each other. Therefore, they might be similar with respect to the measurement timestamps. We further want to add that the regression framework estimates water quality parameters by reflectance values, so no additional data and knowledge of the physical process are employed.

### 5.2. Discussion of the ET’s Feature Importance

To investigate, to understand and to link the hyperspectral data with underlying physical processes of the five water quality parameters, we first start interpreting by focusing on the feature importance. We point out that we discuss the feature importance mainly from a machine learning perspective, and we refer to common characteristics of the water quality parameters. Although the feature importance presented in this paper is one of several definitions, the ET feature importance is internally consistent. This consistency would be lost, when comparing different definitions of a feature importance.

Regarding the feature importance of all five water quality parameters, an important feature (equal to a hyperspectral band) is at a wavelength of approximately 680 nm, which is identified as related to the chlorophyll *a* concentration [40]. From a physical perspective, this peak is produced by the absorption of the red spectrum comparable to the red-edge characteristics of vegetation [11]. In the ET feature importance, this peak is relatively low for green algae, though this water quality parameter contains chlorophyll *a*. Except for green algae, the hyperspectral band at 735 nm is extremely important for the ET regressor.

Generally, bands at the beginning and at the end of the measured spectrum are more dispensable for the ET regression, which could result from, for example, sensor noise. This finding underlines our motivation to ignore the wavelengths between 450 and 470 nm, as well as between 910 and 950 nm as a preceding feature selection step. Another presumption can be derived from the correlation of the feature importance of chlorophyll *a*, green algae, diatoms and turbidity. One possible hypothesis for this correlation is that at the river Elbe, an increasing chlorophyll *a* concentration might determine the appearance of more algae, and finally, it might result in worse visibility, and therefore higher turbidity. The linear correlation between chlorophyll *a* and turbidity is $r^{2}=48\%$. This correlation might support the former hypothesis.

Since the spectral signatures of green algae and chlorophyll *a* demonstrate few common features, we might expect similar shapes of the feature importance plots. As shown in Figure 6, the feature importance of chlorophyll *a* however is closer correlated to the feature importance of diatoms. This characteristic could be unique for the river Elbe. Thus, we need to investigate our findings by conducting measurements of different inland waters.

## 6. Conclusions

In this paper, we evaluated the potential of a regression framework to estimate five water quality parameters with hyperspectral data. The regression framework was applied on measured data. We described the measurement setup during the Elbe field campaign and the three applied sensor systems: the Cubert hyperspectral sensor, the multi-sensor system Biofish and the fluorometer PhycoSens. Two distinct preprocessing methods, PCA and min-max scaling, were included in the regression framework. A detailed evaluation of the regression performance with either one of the preprocessing methods or without preprocessing for each regression model and water quality parameter was presented. Furthermore, we visualized the regression results and the measured values along the shape of the Elbe.

As demonstrated, most of the selected machine learning models were able to handle the high-dimensional data well and were able to estimate water quality parameters such as algae species, turbidity or CDOM based on this data. In this context, machine learning provides a data-driven and well-chosen alternative to the commonly-applied method using feature-engineering such as band-ratios. We conclude that the regression models ANN, SVM and ET were most valuable with respect to the underlying study.

Consequently, we will approach further adjustments. The selection of the regression models included in the framework can be reduced to the most efficient models. We can transfer additional preprocessing techniques that were applied successfully in other areas of hyperspectral remote sensing. Furthermore, we can think about the application of deep learning models [37].

Nevertheless, the modification of the methodological aspects would only improve the already strong performance of the regression framework. We conclude that this contribution instead represents an initial step towards a generic approach. The ability to apply hyperspectral data to perform an area-wide estimation of water quality parameters is demonstrated in this first step. Future investigations will focus on the adaption of the regression framework in regards to applications on several types of inland waters and the area-wide estimation of water quality parameters. Necessary prerequisites in this context are (a) measurements in various inland waters with the applied sensor setup to sample sufficient data, (b) enhancements of the regression framework for this generalization and (c) applications of the framework to multispectral ocean color satellite data [41].

## Figures and Tables

**Table 1:** Number of datapoints per water quality parameter as the target variable and dataset.

Water Quality Parameter	Number of Datapoints		
	Full Dataset	Training Subset	Test Subset
CDOM	802	240	562
Chlorophyll *a*	1035	339	696
Green algae	1028	336	692
Diatoms	1012	332	680
Turbidity	802	240	562

**Table 2:** Regression results for the estimation of CDOM, chlorophyll *a*, green algae, diatoms and turbidity. The **bold** values represent the best regression results, respectively. The RMSE is given in the respective units of the target variables. After applying min-max scaling, the RMSE is unitless.

Variable	Model	Baseline		with PCA		with Scaling	
		$R^{2}$ in %	RMSE	$R^{2}$ in %	RMSE	$R^{2}$ in %	RMSE
CDOM	Linear	74.3	1.05	83.2	0.85	74.3	0.12
	PLS	84.9	0.81	83.2	0.85	84.9	0.09
	RF	82.4	0.87	91.4	0.61	82.4	0.10
	ET	86.2	0.77	**94.6**	**0.48**	86.3	0.09
	AdaBoost	80.0	0.93	91.9	0.59	79.9	0.11
	GB	80.1	0.93	91.2	0.61	80.0	0.11
	k-NN	85.5	0.79	85.3	0.80	83.0	0.10
	SVM	**91.5**	**0.61**	91.2	0.61	85.6	0.09
	ANN	87.2	0.74	50.8	1.44	**93.7**	**0.06**
	SOM	85.8	0.78	83.5	0.84	83.0	0.10
Chlorophyll *a*	Linear	70.2	18.45	75.5	16.70	70.2	0.14
	PLS	73.5	17.38	75.5	16.70	73.5	0.13
	RF	76.5	16.36	88.7	11.35	76.6	0.12
	ET	80.0	15.10	**91.4**	**9.92**	80.0	0.11
	AdaBoost	68.7	18.89	80.0	15.11	66.7	0.14
	GB	76.5	16.36	89.4	10.98	75.5	0.12
	k-NN	76.1	16.51	76.6	16.34	75.4	0.12
	SVM	**88.0**	**11.69**	90.0	10.71	87.6	0.09
	ANN	67.3	19.12	90.5	10.40	**89.3**	**0.08**
	SOM	74.3	17.12	74.7	16.99	71.5	0.13
Green algae	Linear	49.7	14.42	62.3	12.49	49.7	0.18
	PLS	62.6	12.44	62.3	12.49	62.6	0.15
	RF	69.6	11.21	81.6	8.73	69.6	0.14
	ET	73.1	10.55	**87.5**	**7.20**	73.2	0.13
	AdaBoost	60.5	12.78	75.6	10.05	61.7	0.15
	GB	67.0	11.68	80.6	8.95	67.1	0.14
	k-NN	68.8	11.35	68.6	11.40	68.0	0.14
	SVM	**83.1**	**8.36**	79.7	9.18	**76.6**	**0.12**
	ANN	56.8	13.34	81.3	8.79	75.9	0.12
	SOM	64.8	12.06	64.3	12.15	66.2	0.15
Diatoms	Linear	55.0	10.51	62.4	9.60	55.0	0.15
	PLS	58.8	10.06	62.4	9.60	58.8	0.15
	RF	68.2	8.84	81.8	6.68	68.2	0.13
	ET	72.7	8.19	86.4	5.78	72.7	0.12
	AdaBoost	56.7	10.31	76.3	7.62	56.8	0.15
	GB	68.0	8.87	81.5	6.73	67.2	0.13
	k-NN	68.6	8.78	68.6	8.78	67.7	0.13
	SVM	**80.5**	**6.93**	78.2	7.32	**80.3**	**0.10**
	ANN	62.4	9.60	**86.9**	**5.65**	79.8	0.10
	SOM	64.0	9.40	64.2	9.38	63.8	0.14
Turbidity	Linear	45.0	0.44	70.9	0.32	44.0	0.19
	PLS	73.3	0.30	70.9	0.32	73.3	0.13
	RF	68.0	0.33	84.1	0.24	67.7	0.15
	ET	73.6	0.30	**89.9**	**0.19**	73.0	0.14
	AdaBoost	67.6	0.34	85.2	0.23	66.4	0.15
	GB	69.2	0.33	85.5	0.22	69.3	0.14
	k-NN	72.5	0.31	72.8	0.31	70.7	0.14
	SVM	**80.5**	**0.26**	74.3	0.30	73.3	0.13
	ANN	79.9	0.26	88.4	0.20	**80.8**	**0.11**
	SOM	76.0	0.29	71.6	0.31	73.0	0.14
